# Magnetic Nanoparticle-Based DNA Isolation from Refined Soybean Oil: A Cost-Effective Approach for GM Testing

**DOI:** 10.3390/foods14183186

**Published:** 2025-09-12

**Authors:** Yimiao Xia, Mengru Guo, Kunlun Liu, Ying Xin, Fusheng Chen

**Affiliations:** 1 College of Food Science and Engineering, Henan University of Technology, Zhengzhou 450001, China; xiayimiao@haut.edu.cn (Y.X.);; 2 National Engineering Research Center of Wheat and Corn Further Processing, Zhengzhou 450001, China

**Keywords:** DNA extraction, refined soybean oil, magnetic nanoparticles, transgenic detection

## Abstract

Soybean oil has recently emerged as the most widely consumed genetically modified (GM) vegetable oil globally. DNA-based methods offer considerable advantages for monitoring GM-derived products; however, their efficacy strongly depends on the quality and quantity of extracted DNA. Owing to intensive processing, refined oils typically contain extremely low concentrations of severely fragmented DNA, making DNA extraction highly challenging. To address this issue, we introduce an innovative magnetic bead-based DNA extraction protocol specifically tailored to refined soybean oils. Optimal DNA adsorption was achieved using 300 nm carboxyl (-COOH)-modified magnetic beads under optimized conditions, including 1 M guanidine isothiocyanate (GITC) buffer at pH 6.0, combined with ethanol at a 1:1 ratio. Subsequently, we developed a cetyltrimethylammonium bromide (CTAB)-magnetic bead method in which DNA was efficiently transferred from the oil phase to the aqueous phase, concentrated via precipitation, resuspended in GITC buffer, and finally purified using magnetic beads. Comparative evaluations using nested polymerase chain reaction (PCR) and real-time PCR confirmed that this method significantly outperformed traditional CTAB-based methods (CTAB alone, CTAB-hexane) and two representative silica membrane-based extraction kits. Spike recovery experiments further demonstrated its superior efficacy, achieving a DNA recovery rate of 76.37%. The proposed protocol is simple, user-friendly, cost-effective, and highly efficient, markedly reducing reliance on large volumes of organic solvents (e.g., hexane and chloroform) and minimizing the required centrifugation steps. This novel method established an effective approach for DNA extraction from refined vegetable oils, facilitating the development of rapid and reliable GM detection.

## 1. Introduction

Soybeans, renowned for their high protein and oil contents, represent a global agricultural staple, with approximately 74.9% exhibiting biotech traits as of 2024 [1]. Among the genetically modified (GM) soybean varieties, Roundup Ready™ soybean, which confers resistance to glyphosate, has become particularly prominent since its introduction in 1996. Soybeans are predominantly processed into oil, and soybean oil is the second most consumed vegetable oil globally after palm oil [2]. Given the extensive incorporation of GM organisms into food products, food safety concerns have intensified, prompting stringent regulations, including the mandatory labeling and traceability of GM components regulated by the European Union [3], the United States [4], and China (https://www.moa.gov.cn/ztzl/zjyqwgz/zcfg/201007/t20100717_1601302.htm, accessed on 9 August 2025). DNA-based methods are crucial for the detection of transgenic materials, and polymerase chain reaction (PCR) analyses rely heavily on high-quality DNA [5,6]. However, the refining of edible oils poses significant obstacles to DNA extraction due to considerable DNA degradation during processing and the inherently low DNA content resulting from DNA’s water-soluble characteristics, which presents substantial challenges for researchers.

Pauli et al. initially demonstrated DNA isolation from crude and degummed soybean oils using the Wizard DNA extraction method and successfully obtained PCR amplicons exclusively from crude oil [7]. Subsequently, Bogani et al. [8] reported successful amplification of a 500 bp *lectin* gene segment from both crude and degummed oils but encountered difficulty in amplifying the *cp4epsps* gene from degummed oil when employing the “Wizard Magnetic DNA Purification System for Food” kit. Costa et al. [9] significantly advanced the field by detecting *lectin* and *cp4epsps* in deodorized soybean oil using pre-concentration and the NucleoSpin method, marking the first documented successful extraction of DNA from refined soybean oil. Additional methods reported include cetyltrimethylammonium bromide (CTAB) [10,11], DNeasy Plant Mini Kit [10,12], and NucleoSpin Plant II Kit [13], all of which effectively isolate DNA from refined soybean oil. Among these methods, CTAB utilizes a physicochemical mechanism, whereas the DNeasy Plant Mini Kit (protocol: cell lysis → precipitation of proteins, polysaccharides, and other impurities → mixing DNA-containing supernatant with ethanol → DNA specifically binds to the silica membrane → DNA washing → DNA elution) and NucleoSpin (protocol: cell lysis → DNA specifically binds to the silica membrane → DNA washing → DNA elution) methods rely on spin-column purification. In contrast, the Wizard DNA extraction method (protocol: cell lysis → DNA adsorption onto magnetic beads → magnetic bead separation → magnetic bead washing → DNA elution from the beads) uses magnetic beads to facilitate DNA extraction. Despite these advancements, DNA extraction from refined soybean oil remains a significant global research challenge.

Recently, magnetic nano- and microparticles have gained widespread adoption as solid supports for nucleic acid extraction, purification, and concentration with numerous commercially available options [14]. Typically, these magnetic particles comprise a supermagnetic core, protective coating, and functionalized surfaces [15]. The supermagnetic core enables separation without the use of centrifugation, while surface functionalization allows specific interactions and the capture of diverse biomolecules, including nucleic acids [16,17]. Moreover, their large surface area enhanced biomolecular adsorption efficiency. Magnetic beads have been successfully employed for DNA extraction from diverse matrices, including river water [18], environmental samples such as wastewater effluent, activated sludge, freshwater river, and soils [19], *Escherichia coli* [20], and blood [21]. Notably, magnetic bead-based methods generally demonstrate superior DNA extraction efficiency compared to spin-column-based methods, which is particularly advantageous for samples subjected to extensive processing [15]. However, the application of magnetic bead-based DNA extraction from refined edible oils is scarcely documented, and only a few kit-based approaches (e.g., Wizard DNA extraction kit) employ this methodology.

Therefore, this study aimed to develop a novel magnetic bead-based DNA extraction method specifically tailored to refined soybean oil. By dissociating magnetic beads from proprietary commercial kits, this approach maintains high extraction efficiency while significantly reducing economic costs. The optimized protocol established herein not only enhances GM detection capabilities but also provides a robust methodological reference for PCR-based analytical applications, including adulteration detection and traceability analysis of vegetable oils.

## 2. Materials and Methods

### 2.1. Samples

Transgenic soybean oil (CHN grade 1) and non-transgenic soybean oil (CHN grade 1) were purchased from retail stores. Certified reference materials (CRMs) of soya seed powder with different mass fractions of GTS 40-3-2 soybean (<0.09 g/Kg-BF410ap, >985 g/Kg-BF410bp), certified by the Joint Research Centre of the European Commission (Geel, Belgium), were purchased from Fluka (Buchs, Switzerland) and used as the standards. -COOH-modified magnetic beads (magnetic core Fe_3_O_4_, shell SiO_2_, 10 mg/mL, surface carboxyl group content 200 μmol/g magnetic beads, average diameter 100/300/500 nm) and -OH-modified magnetic beads (magnetic core Fe_3_O_4_, shell SiO_2_, 10 mg/mL, nucleic acid-binding ability >20 μg DNA/mg magnetic beads, average diameter 100/300/500 nm) were purchased from BEAVER (Suzhou, China).

### 2.2. DNA Extraction from Soybean Oil by Magnetic Bead Method

The DNA extraction procedure for soybean oil consisted of several sequential steps:

(1) Cell lysis and DNA transfer to the aqueous phase:

Initially, 25 mL of pre-warmed (65 °C) CTAB extraction buffer [5% (*w*/*v*) CTAB, 1.4 M NaCl, 100 mM Tris-HCl, 20 mM ethylenediaminetetraacetic acid disodium salt (EDTA-2Na), 1% (*v*/*v*) Tween-20, 1% (*w*/*v*) polyvinylpyrrolidone 40, pH 8.0] was added to 30 mL of homogenized soybean oil. The mixture was subsequently mixed at 45 rpm for 3 h using an MX-RL-Pro LCD Digital Rotator (DLAB Scientific, Beijing, China). The samples were then centrifuged at 12,000× *g* for 10 min at 20 °C. The upper oily phase (supernatant) was carefully removed. To facilitate oil removal from the emulsion layer, samples were demulsified by incubation in a water bath at 70 °C for 20 min. An additional 30 mL of homogenized soybean oil was added to the lower CTAB buffer layer, and the mixing, centrifugation, and supernatant removal steps were repeated, resulting in a total oil volume of 60 mL.

(2) DNA precipitation and resuspension:

Acryl carrier solution (Solarbio Life Sciences, Beijing, China) was gently added to the lower aqueous phase at a ratio of 5 µL per 1 mL of sample, followed by the addition of 1/10 volume of 3 M sodium acetate (pH 5.2). An equal volume of isopropanol was then mixed with the solution, and the mixture was incubated overnight at −20 °C to precipitate DNA. The sample was centrifuged at 16,000× *g* for 20 min at 4 °C, the supernatant was discarded, and the pellet was resuspended in 1 mL of a designated “specific solution”, optimized in Section 2.7, using a VORTEX1 mixer (IKA-Labortechnik, Stauffen, Germany) before transferring it to a new 2 mL tube.

(3) Oil removal from DNA:

To remove residual oil, 800 μL chloroform–isoamyl alcohol (24:1, *v*/*v*) was added to the resuspended sample, followed by centrifugation at 12,000× *g* for 10 min. The aqueous supernatant was carefully transferred to a new 2 mL tube for further analysis.

(4) Binding of DNA to magnetic beads:

Next, 0.2 mg of “specific magnetic beads” were added to the aqueous DNA solution, followed by mixing with “specific volume” of ethanol/isopropanol. The mixture was gently inverted several times by hand and incubated for 10 min to facilitate DNA binding.

(5) DNA washing:

The clear liquor was pipetted out after the mixture was separated using a Magnetic Separator Stand (BEAVER, Suzhou, China). Subsequently,1 mL of 70% ethanol (*v*/*v*) was added, mixed gently by manual inversion, and separated again using a Magnetic Separator Stand. This washing step was repeated twice to ensure thorough removal of impurities.

(6) DNA elution:

Magnetic beads were air-dried at room temperature for 3~5 min before use. Subsequently, DNA was eluted by adding 100 μL of TE buffer (10 mM Tris, 1 mM EDTA-2Na, pH 8.0), thoroughly mixed using a VORTEX1 mixer, and incubated at 60 °C for 10 min. The beads were then separated using a Magnetic Separator Stand until the TE buffer was clear. Finally, the eluted DNA solution was transferred into a new 600 μL tube and stored at −20 °C until further use.

### 2.3. DNA Extraction from Soybean

DNA extraction from soybean powder was performed using a previously reported method [22].

### 2.4. Determination of the Yield and Purity of DNA Extracted from Soybeans

For DNA extracted from soybeans, the DNA yield was spectrophotometrically determined by measuring the absorbance at 260 nm, and purity was confirmed by measuring the absorbance ratio at 260 nm and 280 nm (A_260/280_ ratio) using a Nanodrop 2000 spectrophotometer (Thermo Scientific, Waltham, MA, USA).

### 2.5. Real-Time PCR

Oligonucleotide primers targeting *lectin* (GenBank: K00821.1) and *cp4epsps* (GenBank: AB209952.1) were used to evaluate the DNA yield of soybean oils. The primer pairs 5′-ACGGCACCCCAAAACCCTC-3′ and 5′-GCGAAGCTGGCAACGCTAC-3′ (94 bp) were used in the q-Lectin amplification, whereas 5′-CAACCACGTCTTCAAAGCAAGT-3′ and 5ʹ-GGAAGGGTCTTGCGAAGGA-3′ (92 bp) were used for q-35S amplification system [23]. The primers were synthesized by Sangon Biotech (Shanghai, China).

Each 20-μL reaction mixture contained isolated DNA (5 μL for soybean oil and 1 μL for soybean seeds), 1× TB Green™ Premix Ex Taq™ II (Tli RNaseH Plus) (TaKaRa Bio, Shiga, Japan), 1× ROX reference dye (TaKaRa Bio, Shiga, Japan), and 150 nM of each primer. Assays were performed in an ABI QuantStudio3 Real-Time PCR System (Applied Biosystems, Foster City, CA, USA) under the following conditions: initial preheating at 95 °C for 10 min, followed by 40 cycles at 95 °C for 15 s and 60 °C for 1 min, with a single fluorescent reading at the end of each cycle. To check for specificity, a melting curve analysis (95 °C for 15 s, 60 °C for 1 min, and 95 °C for 15 s) was performed after the amplification reaction, during which fluorescence measurements were taken in approximately 0.3 °C increments from 60 °C to 95 °C. A no-template and negative control were included in each assay to ensure the absence of contamination.

Standard curves corresponding to q-Lectin and q-35S were generated using >985 g/Kg-BF410bp soybean extracts. A 10-fold serial dilution (500, 50, 5, 0.5, and 0.05 ng/μL) were prepared, and the quantification cycle (Cq) values were determined in triplicate for each dilution. A standard curve was generated based on the DNA yield and corresponding Cq values [Cq = a × l g (DNA yield) + b]. Parameters such as efficiency (E) and correlation coefficient (R^2^) were derived.

### 2.6. Optimization of Magnetic Bead Type

Variations in bead particle size were measured to evaluate the DNA adsorption capacity of the magnetic beads. Magnetic beads (20 μL; 10 mg/mL; BEAVER, Suzhou, China) were diluted to 10 µg/mL using an ethanol/guanidine isothiocyanate (GITC) mixture (1:1, *v*/*v*). The mixture was thoroughly homogenized using a VORTEX1 mixer and ultrasonicated using a KQ5200DE ultrasonic cleaner (SHUMEI, Shanghai, China). Additionally, 20 μL of magnetic beads (10 mg/mL) were combined with 1 μL of soybean DNA (2616 ng/μL) and 1 mL of GITC solution (1 mol/L, pH 6.0). The solution was vortexed thoroughly and ethanol (1 mL) was added. The mixture was then incubated for 10 min with several manual overturns. Subsequently, the mixture was diluted to a final concentration of 10 µg/mL using 17.979 mL ethanol/GITC mixture (1:1, *v*/*v*). The particle sizes of the magnetic beads were analyzed using a BeNano 90 Zeta instrument (BetterSize, Dandong, China). Each sample was analyzed three times at room temperature.

DNA recovery rate test in the aqueous phase: 20 μL of magnetic beads (10 mg/mL), 1 μL of soybean DNA (2616 ng/μL), and 1 mL of GITC solution (1 mol/L, pH 6.0) were vortexed thoroughly, followed by the addition of 1 mL ethanol. The mixture was incubated for 10 min with intermittent manual inversion. DNA washing and elution procedures were performed as described in Section 2.2. DNA concentration was measured using a NanoDrop 2000 spectrophotometer. DNA recovery rate (%) was calculated using the following formula: (DNA yield × 100 μL × 100%)/2616 ng.

DNA recovery rate test in the oil phase: DNA extraction was performed following the procedure described in Section 2.2, with modifications specifying the use of non-transgenic soybean oil (CHN grade 1) adding 175 ng of DNA extracted from >985 g/Kg-BF410bp soybean, GITC solution (1 M, pH 6.0), and 1 mL ethanol as the respective oil material, “specific solution”, and “specific ethanol/isopropanol volume”. The two optimized magnetic bead types identified in this section were used. DNA yield was quantified using real-time PCR targeting the q-35S amplification system. DNA recovery rate (%) was calculated as (DNA yield × 100 μL × 100%)/175 ng.

### 2.7. Optimization of Magnetic Adsorption System

Following the identification and optimization of “specific magnetic beads”, DNA extraction from soybean oil was performed, as detailed in Section 2.2. The magnetic adsorption system, defined by the “specific solution” in step 2 and the “specific ethanol/isopropanol volume” in step 4, was optimized based on the DNA yield as measured via real-time PCR (q-35S amplification system).

For optimization of the “specific solution”, comparative evaluations were conducted between 1 M GITC and 13.5% PEG-3 M NaCl. GITC solutions at concentrations of 0, 1, 2, 3, and 4 M were systematically analyzed to determine the optimal GITC concentration. The pH values of the GITC solutions were individually tested at 5.5, 6.0, 6.5, 7.0, 7.5, and 8.0, to determine the most effective pH value.

To optimize the “specific ethanol/isopropanol volume”, the comparative effectiveness of ethanol and isopropanol was evaluated. The volume ratios of ethanol/isopropanol to GITC solution tested were 1:2, 1:1, and 3:2 to identify the optimal proportion that yielded the highest DNA recovery.

### 2.8. DNA Extraction from Soybean Oil Using Different Methods

DNA was isolated from soybean oil (CHN grade 1) by five different methods.

#### 2.8.1. CTAB–NucleoSpin Food Kit Method

DNA pellets obtained from step 2, described in Section 2.2 were resuspended in lysis buffer provided by the NucleoSpin Food Kit (Macherey–Nagel, Düren, Germany). The subsequent extraction steps were performed according to the manufacturer’s protocol.

#### 2.8.2. CTAB-Magnetic Bead Method

As outlined in Section 2.2, the specific solution components were adjusted as follows: GITC solution (1 M, pH 6.0), carboxyl (-COOH)-modified magnetic beads (300 nm), and 1 mL of ethanol were used as the “specific solution”, “specific magnetic beads”, and “specific volume of ethanol/isopropanol”, respectively. All other procedures followed the methodology described in Section 2.2.

#### 2.8.3. CTAB–Hexane Method

Initially, 30 mL soybean oil was mixed with 25 mL hexane at 45 rpm for 3 h using an MX-RL-Pro LCD Digital Rotator. Subsequently, 25 mL of preheated (65 °C) CTAB extraction buffer was added, and the mixture was mixed at 45 rpm for an additional 3 h. After centrifugation at 12,000× *g* for 10 min at 20 °C, the upper organic phase was discarded. The aqueous phase was supplemented with acryl carrier (0.005 volumes), sodium acetate solution (3 M, pH 5.2; 0.1 volume), and an equal volume of isopropanol. The mixture was incubated at −20 °C for 1 h to facilitate DNA precipitation. Following centrifugation at 16,000× *g* for 10 min at 4 °C, the supernatant was carefully removed. The resulting pellet was washed twice with 1 mL 70% ethanol and dried at 20 °C. Finally, the pellet was resuspended in 100 μL TE buffer, then this TE buffer was used to suspend the pellet in second tube to ensure a total processed oil volume of 60 mL.

#### 2.8.4. CTAB Method

First, 30 mL of soybean oil was mixed with 25 mL of preheated (65 °C) CTAB extraction buffer at 45 rpm for 3 h. After centrifugation at 12,000× *g* for 10 min at 20 °C, the upper oily phase (excluding the emulsion layer) was removed. An additional 30 mL of soybean oil was added, mixed for 3 h, and centrifuged. After removing the upper oily phase (excluding the emulsion layer), 25 mL of chloroform–isoamyl alcohol (24:1, *v*/*v*) was added, mixed for 2 min, and centrifuged at 12,000× *g* for 10 min. The upper aqueous phase was transferred to a fresh 100 mL tube, supplemented with acryl carrier (0.005 volumes), sodium acetate (3 M, pH 5.2, 0.1 volumes), and an equal volume of isopropanol, and incubated at −20 °C for 1 h to precipitate DNA. After centrifugation at 16,000× *g* for 10 min at 4 °C, the supernatant was discarded. The DNA pellet was washed twice with 1 mL 70% ethanol, dried at 20 °C, and resuspended in 100 μL TE buffer.

#### 2.8.5. Resin Based Oil DNA Extraction Kit Method

Genomic DNA was extracted using a resin-based oil DNA extraction kit (DINGGUO, Beijing, China), according to the manufacturer’s protocol. Briefly, 30 mL of soybean oil, 22.5 mL of solution A (chloroform), and 15 mL of solution B (1.4 M NaCl) were combined in a 100 mL centrifuge tube and agitated at 45 rpm for 3 h. Following phase separation by centrifugation at 12,000× *g* for 10 min at 20 °C, the upper oily phase was carefully discarded. Subsequently, 50 μL of solution C (resin) was added to the lower aqueous phase and mixed thoroughly via manual inversion. The mixture was centrifuged at 8000× *g* for 5 min. After removing the supernatant, 800 μL of solution B was added to the pellet and gently mixed. The pellet solutions from two separate tubes were pooled into a single 2 mL microcentrifuge tube to ensure a combined oil sample volume of 60 mL. Following an additional centrifugation step at 8000× *g* for 5 min, the supernatant was removed, and the pellet was washed twice with 1 mL of solution D (70% ethanol). The pellet was then air-dried at 20 °C and resuspended in 100 μL TE buffer. The suspension was incubated in a water bath at 55 °C for 5 min and then centrifuged at 12,000× *g* for 2 min. The resulting supernatant, containing purified DNA, was transferred to a new 600 μL microcentrifuge tube and stored at −20 °C for subsequent analysis.

### 2.9. Conversion of DNA Mass Concentration in Soybean Oil

This study quantified DNA extracted from soybean oil via real-time PCR, reporting concentrations in ng/μL (in TE buffer). DNA was isolated from 60 mL of soybean oil and finally dissolved in 100 μL TE buffer. DNA concentration was converted to reflect the original oil matrix using the following formula:

$$C_{2}\;(ng/mL)=\frac{C_{1}\times 100}{60}$$

where *C*_1_ represents the DNA mass concentration determined by real-time PCR (ng/μL); *C*_2_ denotes the DNA mass concentration of the original oil.

Since the haploid soybean genome has a mass of approximately 1.13 pg (https://cvalues.science.kew.org/search, accessed on 9 August 2025), the DNA mass concentration (ng/mL) can be converted to DNA copy number per milliliter (copies/mL) using the following formula:

$$C_{3}\;(copies/mL)=\frac{C_{2}\times 1000}{1.13}$$

where *C*_2_ represents the DNA mass concentration in the original oil (ng/mL); *C*_3_ denotes the DNA mass concentration in the original oil in terms of copy number.

### 2.10. Two-Round Nested Qualitative PCR

As the quantity of DNA in the refined oils was limited, two-round nested PCR was performed to improve the sensitivity of qualitative PCR. Briefly, the total reaction volume of both the first- and second-round nested PCR was 25 μL, and the reaction mixture contained 1 × Premix Taq^TM^ (TaKaRa Bio Inc., Beijing, China). The first-round PCR mixture contained 40 nM of primer and 5 μL of template DNA, whereas the second-round PCR mixture contained 400 nM of primer and 3 μL of the PCR product from the first-round PCR. Thermal cycling was performed in a Techne TC-412 Thermal Cycler (Staffordshire, UK) under the following conditions: initial denaturation at 95 °C for 5 min, followed by 35 cycles of denaturation at 94 °C for 30 s, annealing for 30 s at specific temperatures (Table 1), and elongation at 72 °C for 30 s, followed by a final extension at 72 °C for 10 min. All samples were analyzed in triplicate, and no-template and positive controls were included to ensure the absence of contamination.

### 2.11. Agarose Gel Electrophoresis

Amplification products were separated by electrophoresis using a 2.5% agarose gel (Sangon Biotech, Shanghai, China) containing 4S green plus nucleic acid stain (Sangon Biotech, Shanghai, China) in 1 × TAE buffer. Images were visualized and recorded using a UV Bio-Rad Gel Doc 2000 system (BioRad, Hercules, CA, USA) and the associated Quantity One software version 4.4.

### 2.12. Evaluation of DNA Recovery Rates Across Different Extraction Methods

To evaluate DNA recovery rates, 1120 ng of DNA extracted from >985 g/Kg-BF410bp soybean was thoroughly mixed with 60 mL of non-transgenic soybean oil. DNA was subsequently extracted using the five methods described in Section 2.8. The extracted DNA was quantified by real-time PCR using a q-35S amplification system. DNA recovery rates were calculated using the following formula: (DNA yield × 100 μL × 100%)/1120 ng.

### 2.13. Data Analysis

Each sample was examined in triplicate. The Independent Samples *t*-Test was used to analyze significant differences between two independent means. One-way ANOVA was employed to examine significant differences among three independent means. All analyses were performed using SPSS software version 21.0 (IBM Analytics, Armonk, NY, USA).

## 3. Results and Discussion

### 3.1. Design of DNA Extraction Method

**Figure 1:**
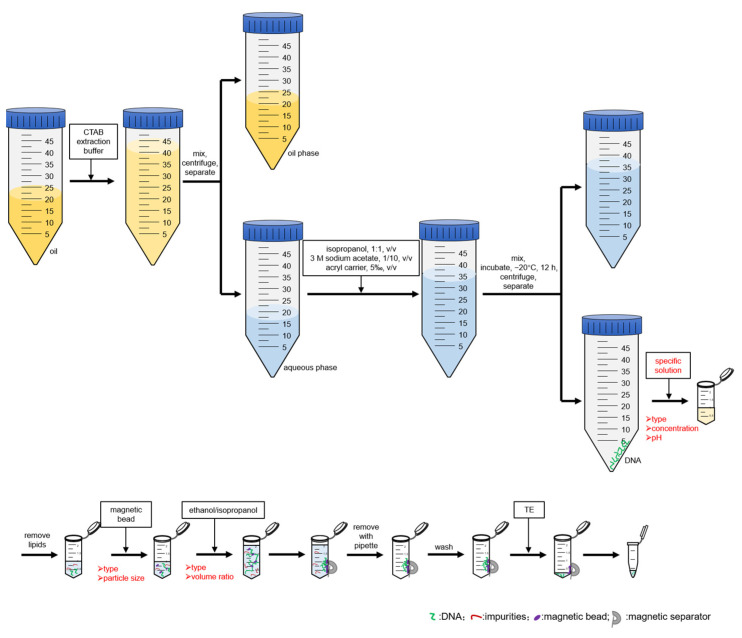
Schematic diagram of the protocol for the CTAB-magnetic bead method. Note: the steps described in red font would be optimized in this study.

Previous studies [13,24] employed high-speed centrifugation to isolate pellets from refined oils for DNA extraction. However, in the current study, high-speed centrifugation did not yield pellets from grade 1 refined soybean oil, likely because of the extensive refinement process involved. Following centrifugation, pellet formation was observed only in crude and cold-pressed sunflower oils, but not in refined oils [25]. Consequently, we developed an alternative DNA extraction strategy that involved the phase transfer of DNA from refined oil to an aqueous phase, followed by precipitation, resuspension, and purification of DNA from the aqueous phase (Figure 1).

To optimize DNA recovery, an aqueous extraction buffer was formulated with 5% (*w*/*v*) CTAB, and the water-to-oil volume ratio was set as 5:6 [26]. When emulsion layers formed during phase separation, heating at 75 °C was applied for demulsification. This method significantly reduces the reliance on organic solvents (e.g., chloroform and n-hexane), minimizing the potential risks of DNA degradation associated with these reagents.

A notable innovation of this study was the incorporation of magnetic beads for DNA extraction from refined oils. The screening of magnetic beads was conducted based on surface functionality and particle size to enhance binding efficiency. Further optimization included the assessment of the composition, concentration, pH, and polarity of the magnetic adsorption buffer. After the type of magnetic beads and its corresponding composition of adsorption system was optimized, DNA precipitation from aqueous phase was resuspended in the adsorption buffer (Figure 1). Following magnetic beads addition and DNA binding, a magnetic separator is used to collect the magnetic beads. The clear solution was pipetted to remove impurities. Given that residual salts can negatively impact PCR sensitivity [27], a washing step using 70% ethanol was performed prior to elution. DNA was efficiently eluted in TE buffer at 60 °C for 5 min. This magnetic bead-based approach markedly reduced the need for centrifugation and improved the sample handling efficiency.

### 3.2. Identification of Magnetic Bead Type

Magnetic nanoparticles used for nucleic acid extraction can be categorized into positively and negatively charged nanoparticles based on their surface charge properties [28]. Nucleic acids inherently possess strong negative charges owing to their phosphate backbone, facilitating electrostatic adsorption onto positively charged magnetic particles. Although positively charged particles exhibit greater adsorption capacity, they often present significant challenges during nucleic acid desorption [29]. Although increasing the solution pH or anion concentration can improve elution efficiency, the overall DNA recovery typically remains suboptimal, and high salt concentrations may adversely affect subsequent PCR assays [19]. Therefore, negatively charged magnetic particles were selected for DNA extraction in this study.

**Figure 2:**
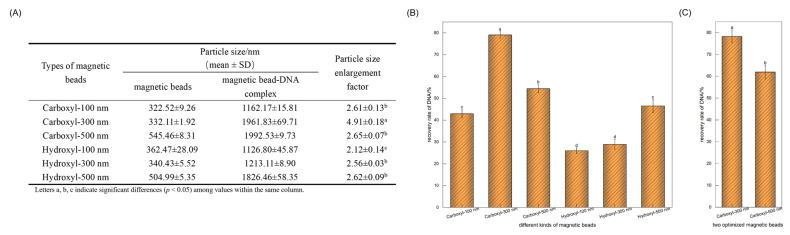
Screening of magnetic bead types. (**A**) Change in particle size of magnetic beads before and after DNA binding; (**B**) Effect of different types of magnetic beads on DNA recovery rate in the aqueous phase; (**C**) Effect of two optimized magnetic beads on DNA recovery rate in the oil phase. Note: different superscript letters indicate significant differences (*p* < 0.05).

The negatively charged magnetic particles evaluated in this study were functionalized with either -COOH or hydroxyl (-OH) groups. As shown in Figure 2A, the -COOH-modified magnetic beads exhibited a more significant increase in particle size following DNA adsorption and a higher DNA recovery rate than the -OH-modified beads. These results suggest that -COOH-modified magnetic beads are more suitable for soybean DNA extraction.

Smaller magnetic beads typically have larger specific surface areas, which enhances their adsorption capacity. However, excessively small beads exhibit reduced magnetic responsiveness [15]. Considering that bead size significantly affects the biomolecule detection limit [30], we carefully evaluated the optimal bead diameter for refined soybean oil genomic DNA extraction. As illustrated in Figure 2B, -COOH-modified magnetic beads with a diameter of 300 nm yielded the highest DNA recovery rate. When comparing 300 nm and 500 nm -COOH-modified beads in the DNA extraction procedure described in Section 2.2, the 300 nm beads demonstrated a superior recovery efficiency (Figure 2C). Therefore, 300 nm -COOH-modified magnetic beads were identified as the optimal selection.

### 3.3. Identification of Magnetic Adsorption System

Electrostatic repulsion exists between the negatively charged -COOH-modified magnetic particles and DNA, hindering the direct adsorption of DNA onto these particles. To overcome this limitation, solution-mediated driving forces are required [29]. Commonly employed mediators include chaotropic salts (e.g., guanidine hydrochloride and GITC) and polyethylene glycol (PEG)-NaCl solutions [27]. In this study, GITC and PEG-NaCl solutions were selected to evaluate the adsorption efficiency of negatively charged -COOH-modified magnetic particles.

**Figure 3:**
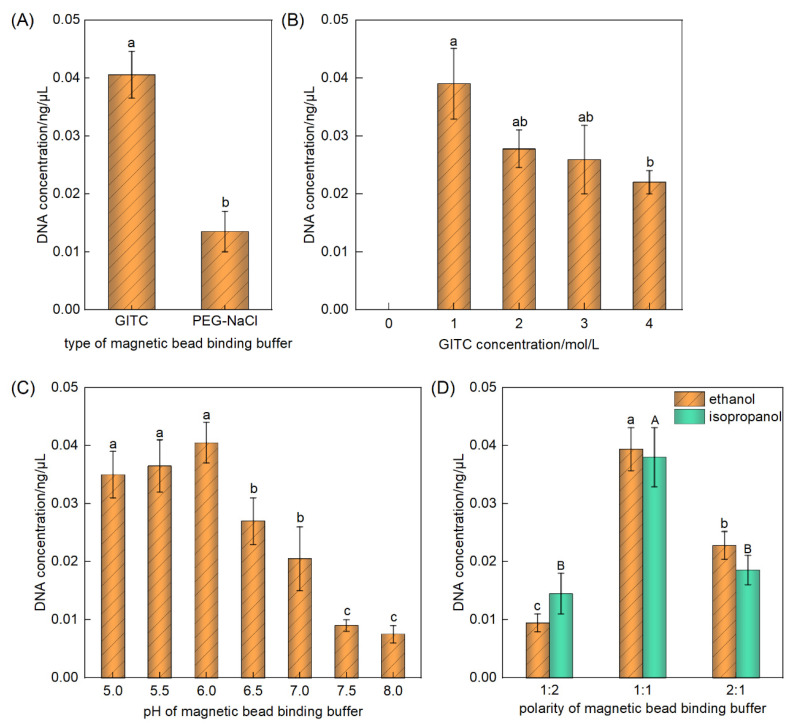
Optimization of the composition of the carboxyl-modified magnetic bead adsorption system. (**A**) Optimization of the magnetic bead binding buffer type; (**B**) Optimization of the concentration of GITC; (**C**) Optimization of the pH value of GITC buffer; (**D**) Optimization of the polarity of magnetic bead binding buffer. Note: different superscript letters indicate significant differences within the group (*p* < 0.05).

As a chaotropic salt, GITC employs a two-step process to facilitate the adsorption of DNA onto the magnetic beads. The first step is GITC can disrupt the electrostatic repulsion between DNA and negatively charged magnetic particles, as well as break the hydration layers surrounding both the DNA and particle surfaces, facilitating their direct interactions [31]. The second step is GITC promotes DNA adsorption onto particle surfaces through the mediation of hydrogen bonds and van der Waals forces [32]. In contrast, PEG solutions induce conformational changes in DNA molecules from an extended, relaxed state to a condensed, supercoiled form, a transition further enhanced by Na^+^ ions [27]. The long-axis length of condensed DNA is approximately 1000-fold shorter than that of extended DNA. In the condensed state, DNA molecules display significantly reduced electrostatic repulsion owing to the internalization of negatively charged phosphate groups. This structural change facilitates effective DNA interactions with magnetic particles. Additionally, PEG disrupts the hydration shells around DNA and particles, enabling ion-bridging interactions and subsequent DNA enrichment [31]. Nevertheless, the PEG-NaCl binding buffer exhibited significantly inferior performance compared to the GITC buffer in capturing soybean oil-derived DNA using -COOH-modified magnetic beads (Figure 3A). Consequently, the GITC buffer was selected for further optimization.

The salt concentration critically influences the adsorption efficiency by modulating the interaction strength between the DNA and functional groups on the magnetic bead surfaces. Higher salt concentrations generally facilitate the precipitation and adsorption of larger DNA fragments more efficiently, whereas excessively low salt concentrations impede DNA adsorption [15,33]. Therefore, DNA extraction efficiency varied significantly with different salt concentrations. As shown in Figure 3B, the optimal DNA extraction efficiency for soybean oil samples was achieved using an initial GITC concentration of 1 M, resulting in a final concentration of 0.5 M in the adsorption system. Previous studies have reported varying optimal GITC concentrations depending on the DNA source and fragment length: 3 M for plasma samples [34], 2.4 M for possum excreta [35], and 1.45 M for Pasteur pipette samples [36]. Compared to DNA from plasma samples, possum excreta, and Pasteur pipette samples, DNA from refined oil exhibited severe degradation and shorter fragment lengths. Consequently, the optimal GITC concentration was the lowest. These discrepancies suggest that the optimal GITC concentration is closely related to the specific length and structural characteristics of target DNA.

In addition to promoting DNA adsorption, GITC effectively denatures proteins and facilitates the dissociation of nucleoprotein complexes, thereby enhancing DNA purity [37]. Consequently, GITC is commonly incorporated into lysis buffers used for DNA extraction. Although several studies have combined cell lysis and magnetic bead adsorption into a single step [35,36,38], limited attention has been given to optimizing the pH of adsorption systems specifically for magnetic bead-based DNA capture. Typically, a high-salt, low pH environment is favorable for DNA adsorption [29]. Consistent with this, our results indicated that an adsorption system with a pH of 6 was optimal for extracting DNA from soybean oil (Figure 3C).

Furthermore, the addition of appropriate concentrations of ethanol or isopropanol reduces system polarity, diminishing the electrostatic repulsion between DNA and negatively charged magnetic beads, thereby enhancing the adsorption efficiency [32]. However, excessive alcohol concentrations promote co-adsorption of protein contaminants, ultimately compromising DNA purity [15]. As shown in Figure 3D, the optimal DNA extraction efficiency from soybean oil was achieved using ethanol as a polarity modifier at a final concentration of 50%. This finding aligns with the variations observed in previous reports using alcohol modifiers: 40% ethanol for possum excreta DNA extraction [35], 30% isopropanol for plasma DNA extraction [34], and 36% isopropanol for Pasteur pipette-derived DNA extraction [36]. The differences in optimal alcohol concentrations were likely associated with the varying levels of protein contaminants in the respective samples.

In summary, the optimized adsorption system composition for DNA extraction from soybean oil utilizing -COOH-modified magnetic beads was established as a 1 M GITC buffer at pH 6.0, mixed in a 1:1 ratio with ethanol.

### 3.4. Validation of the Optimized Magnetic Bead Method

The CTAB method is commonly employed for DNA extraction; however, DNA obtained using this method often demonstrates low purity [12], prompting the development of improved variations, such as the CTAB-hexane method [39]. Recently, silica membrane-based DNA extraction methods, such as the CTAB-NucleoSpin Food Kit and Resin-Based Oil DNA Extraction Kit, have gained widespread adoption owing to their specificity for DNA binding. Additionally, magnetic separation methods, exemplified by the Wizard Magnetic Purification System for Food kit, have been increasingly utilized for DNA extraction. Given that magnetic bead-based methods have rarely been applied for DNA isolation from oils despite their notable advantages, this study independently developed a CTAB-magnetic bead method. To acquire sufficient DNA for PCR and enhance the traceability and sensitivity of GM detection in oil samples, the newly developed method was rigorously compared with the traditional CTAB method, CTAB-hexane method, and two silica membrane-based extraction methods.

The A_260_/A_280_ and A_260_/A_230_ ratios are conventional indicators of DNA purity. Previous studies [9,40,41] have demonstrated that these ratios show no significant correlation with the PCR amplification success in refined oils. Therefore, these purity indicators were not reported in this study. Nested PCR was preferred because of its proven efficacy in amplifying trace DNA from highly processed samples such as refined oils [25,42]. Additionally, nested PCR amplicons were deliberately designed to be <200 bp to maximize amplification success. Real-time PCR was performed using the previously optimized q-35S amplification system established by our research group, which has demonstrated high specificity and sensitivity for detecting the *cp4epsps* gene in refined soybean oil [23].

Nested PCR (Table 2) and real-time PCR (Table 3 and Table 4) results indicated that only the CTAB–NucleoSpin Food Kit method and CTAB-magnetic bead method can extract sufficient DNA for PCR amplification, which means that the DNA extraction efficiency of these two methods is superior to that of the CTAB-hexane, CTAB, and Resin-based Oil DNA Extraction Kit methods. Moreover, the CTAB-magnetic bead method built in this study demonstrated optimal performance based on the extracted DNA yield from refined soybean oil (Table 3 and Table 4). Based on the results of the q-35S quantitative system and the conversion formula described in Section 2.9, the DNA concentration extracted by the CTAB-magnetic bead method was determined to be 56 copies/mL of oil when expressed in terms of copy number.

Neither CTAB nor the CTAB-hexane method successfully extracted PCR-amplifiable DNA from refined soybean oil samples (Table 2, Table 3 and Table 4). The failure of the CTAB method may be attributed to the impurities in the extracted DNA, which inhibit PCR amplification. However, due to the extremely low DNA content in refined oil, this inhibitory effect cannot be overcome by diluting the DNA. The low efficiency of the CTAB method for vegetable oils was consistent with the results of previous studies. For instance, CTAB extraction of DNA from pressed poppy oil resulted in unreliable PCR amplification [43]. Similarly, no PCR amplicons were observed for the *helianthinin* gene when DNA was extracted from refined sunflower oil using the CTAB method [25]. As for the CTAB-hexane method, two key issues arise. Firstly, the prolonged 3 h mixing of soybean oil and hexane may degrade the already limited DNA present in the soybean oil. Secondly, after mixing and centrifuging the mixture of soybean oil, hexane, and CTAB extraction buffer, the aqueous phase—which contained the target DNA—settled at the bottom layer. However, this same layer also contained denatured proteins and other impurities. Consequently, the extracted DNA not only exhibited a lower mass concentration but also higher impurity levels, directly inhibiting PCR amplification.

Among the silica membrane-based methods, only the CTAB-NucleoSpin Food Kit method successfully isolated amplifiable DNA from refined soybean oil, whereas the resin-based Oil DNA Extraction Kit method did not (Table 2, Table 3 and Table 4). These two methods differ notably in the protocols used for DNA transfer from the oil to the aqueous phase. Specifically, the CTAB-NucleoSpin Food Kit directly mixed the oil samples with CTAB buffer for 3 h, followed by centrifugation and incubation at 70 °C for demulsification and oil phase removal. Conversely, the Resin-based Oil DNA Extraction Kit employs chloroform, NaCl, and oil mixing for 3 h, followed by centrifugation to separate and remove the oil phase. Prolonged exposure to organic solvents such as hexane or chloroform can lead to further DNA degradation, possibly explaining why the resin-based Oil DNA Extraction Kit failed to extract amplifiable DNA from refined soybean oil in this study.

Both the CTAB-NucleoSpin Food Kit and CTAB-magnetic bead methods were effective for DNA extraction from refined soybean oil (Table 2, Table 3 and Table 4). The CTAB-NucleoSpin Food Kit employs a spin-column-based technique, wherein the DNA adsorption efficiency of silica membranes is relatively low [44], thereby directly reducing the DNA extraction efficiency. In contrast, this study systematically evaluated different magnetic bead types and optimized the composition of the magnetic bead adsorption system to ensure a high DNA-binding efficiency. Sterile distilled water has been reported to elute DNA from magnetic beads [18,36,45]; however, TE buffer has demonstrated a superior elution efficiency [46]. Therefore, in this study, we used the TE buffer to ensure complete DNA elution. Moreover, magnetic bead-based methods have shown particularly high efficacy in purifying highly degraded DNA [15], which is typical in refined soybean oil. Consequently, the optimized CTAB-magnetic bead method demonstrated higher DNA extraction efficiency for refined soybean oil than the CTAB-NucleoSpin Food Kit method. The magnetic bead-based method outperformed silica column-based approaches in extracting DNA has also been reported in detecting *T. cruzi* satDNA [15,21].

The oil sample volume utilized in this study was 60 mL, which was significantly greater than the volumes reported in some studies (e.g., 1 mL [42], 3 mL [41], and 6 mL [43]), but lower than others (e.g., 100 mL [26] and 320 mL [11]). Additionally, the DNA concentration extracted in this study (56 copies/mL oil) was higher than some previously reported concentrations (0.25 copy/mL oil [9]) but lower than others (>311 copies/mL oil [26]). Regarding the specific DNA extraction method, the Wizard^®^ Magnetic DNA Purification System for Food kit was employed: lysis Buffer A, lysis Buffer B, precipitation solution, and oil are mixed at a ratio of 2:1:3:40 and aqueous phase was collected after centrifugation, then particles were added to aqueous phase to absorb DNA, followed by washing with ethanol 70% (*v*/*v*) and redissolving in 0.1 × TE buffer [9]. The CTAB DNA extraction method was employed by He et al. [26]: 30 mL of oil was blended with an equal volume of chloroform for 3 h, followed by mixing with 25 mL of 5% CTAB buffer for another 3 h; after centrifugation, the aqueous phase was collected and combined with an equal volume of isopropanol to precipitate DNA overnight; then, DNA was re-dissolved after centrifugation, purified using phenol/chloroform/isoamyl alcohol (25:24:1, *v*/*v*/*v*), and finally re-dissolved in TE buffer. Although the Wizard^®^ Magnetic DNA Purification System for the Food kit method also employs magnetic bead technology, both the operational details for transferring DNA from the oil phase to the aqueous phase and the types of magnetic beads used differ from those applied in this study. In comparison with the CTAB method used by He et al. [26], the DNA extraction method adopted in this research was based on magnetic beads, making the two approaches entirely distinct.

These discrepancies in DNA extraction efficiency likely stem not only from differences in DNA extraction methodologies but also from the variability inherent to the oil samples. The term “refined soybean oil” in the literature refers to oils that have been processed to varying degrees. For instance, when employing the CTAB-hexane method, 500 µL of hexane was added to 1 mL of oil, yielding both liquid and solid phases upon mixing and centrifugation [42]. Conversely, the refined soybean oil used in this study under identical conditions produced only a liquid phase without any solid phase formation. Similarly, several studies [9,24] obtained pellets through high-speed centrifugation at 4 °C, removing the liquid phase, and dipping the tube in liquid nitrogen; however, no such pellet formation occurred in this study under identical conditions. Oils that form a solid phase or pellets are typically less refined and contain higher DNA contents. Conversely, oils that do not yield pellet formation are generally highly refined and contain significantly lower DNA concentrations, posing greater extraction challenges. Due to the lack of detailed information about “refined soybean oil” across various studies and the variations in DNA extraction methods, it is not possible to objectively compare or evaluate the efficiency across different DNA extraction protocols from literature.

To validate the effectiveness of the optimized CTAB-magnetic bead method, spiked blank experiments were conducted (Table 5 and Table 6). The highest DNA recovery rate observed was 76.37%, confirming the method’s effectiveness, although residual DNA loss during extraction was also observed. DNA loss can occur during DNA transfer from the oil phase to the aqueous phase, DNA precipitation by isopropanol, magnetic bead adsorption, or washing steps. This study established a magnetic bead-based method for DNA extraction, in which the efficiency of DNA adsorption onto magnetic beads directly influences DNA extraction recovery. The efficiency of DNA adsorption onto magnetic beads is influenced by factors such as bead size, type, and composition of the adsorption buffer. The selection of magnetic bead parameters was further determined by the length of the DNA fragments and concentration of protein impurities in the system. For example, longer DNA fragments require a higher concentration of GITC in the adsorption buffer [31], whereas a higher level of protein impurities necessitates maintaining a sufficiently high polarity in the system to avoid protein co-precipitation, which would reduce the purity of the extracted DNA [15]. Therefore, prior to establishing the CTAB-magnetic bead method, we optimized the magnetic bead parameters to enhance the DNA adsorption efficiency. However, during the DNA extraction process, DNA loss is unavoidable. We can only improve the methods and their details to minimize DNA loss.

DNA extraction from refined oil continues to pose a significant global challenge, primarily due to the extremely low DNA concentrations, high DNA degradation, and procedural losses during DNA extraction.

### 3.5. Cost Comparison

Both the CTAB-NucleoSpin Food Kit and CTAB-magnetic bead methods successfully yielded positive PCR amplicons from DNA extracted from refined soybean oil. These protocols share identical raw materials and pre-concentration steps, with cost discrepancies primarily due to downstream purification processes. The NucleoSpin Food Kit is priced at approximately 332 USD for 50 units, translating to an extraction cost of 6.64 USD per sample. In contrast, the CTAB-magnetic bead method employs 300 nm -COOH-modified magnetic beads (66.82 USD/mL), requiring 4 μL per extraction (0.27 USD). Additional reagents included 1 mL GITC buffer, 1 mL chloroform-isoamyl alcohol, 3 mL anhydrous ethanol, and 100 μL TE buffer. The overall cost of the magnetic bead-based extraction method is approximately 0.42 USD per sample. The current price provided is for reference only, as it may be affected by fluctuations in reagent costs and other factors. However, by decoupling magnetic beads from commercial kits, the cost of DNA extraction can be significantly reduced compared to using commercial kits.

## 4. Conclusions

Through systematic screening of magnetic bead types, optimization of the bead adsorption system, and integration of DNA pre-concentration with magnetic bead purification, we established a robust and highly efficient CTAB-magnetic bead method for DNA extraction from refined soybean oil. This newly developed protocol demonstrated superior DNA extraction performance compared to conventional methods, including the CTAB-NucleoSpin Food Kit (spin-column-based), CTAB alone, and CTAB-hexane methods. In addition, the established method offers simplicity, cost-effectiveness, and a significant reduction in centrifugation requirements, thereby enabling practical and economical GM testing. Beyond GM detection, this optimized CTAB-magnetic bead method provides a valuable reference for DNA extraction protocols applicable to broader DNA-based analyses, such as detecting oil adulteration and tracing geographical origins.

## Figures and Tables

**Table 1:** Primer pairs used for nested PCR.

No.	Name	AL (bp)	AT (°C)	Sequence (5′–3′)	Round	Reference
1	Lectin-F1	94	58.0	ACGGCACCCCAAAACCCTC	2	[10]
	Lectin-R1			GCGAAGCTGGCAACGCTAC		
	Lectin-F2	160	54.5	CCTCGGGAAAGTTACAA	1	
	Lectin-R2			GGGGCATAGAAGGTGAA		
2	35S-F2	195	55.4	GCTCCTACAAATGCCATCA	2	
	35S-R2			GATAGTGGGATTGTGCGTCA		
	Soybean genome/35S-F1	370	53.6	TTCAAACCCTTCAATTTAACCGAT	1	
	Soybean genome/35S-R1			AAGGATAGTGGGATTGTGCGTC		
3	35S/CTP-F1	99	50.1	GACACGCTGACAAGCTGACTC	2	
	35S/CTP-R1			GGAAATTGGAATTGGGATTAA		
	35S/CTP-F2	169	52.8	ATCCCACTATCCTTCGCAAGA	1	
	35S/CTP-R2			TGGGGTTTATGGAAATTGGAA		

No., number; AL, amplicon length; AT, annealing temperature; Round 2, primers used in the second round; Round 1, primers used in the first round.

**Table 2:** Comparison of DNA extraction efficiencies based on nested PCR.

Name	CTAB–NucleoSpin Food Kit Method	CTAB-Magnetic Bead Method	CTAB–Hexane Method	CTAB Method	Resin Based Oil DNA Extraction Kit Method
Lectin-F1	++	++	/	/	/
Lectin-R1					
Lectin-F2					
Lectin-R2					
35S-F1	++	++	/	/	/
35S-R1					
Soybean genome/35S-F1					
Soybean genome/35S-R1					
35S/CTP-F1	++	++	/	/	/
35S/CTP-R1					
35S/CTP-F2					
35S/CTP-R2					

Note: /, no amplification; ++, >50% positive amplification; the original image is shown in Figure A1.

**Table 3:** Calibration curves targeting *lectin* and *cp4epsps*.

Target	Standard Curve		Accuracy and Efficiency	
	Slope	Intercept	R^2^	E (%)
q-Lectin	−3.237	31.71	0.9984	103.67
q-35S	−3.209	31.89	0.9937	104.94

**Table 4:** Comparison of DNA extraction efficiencies based on real-time PCR.

Methods	Parallel Sample	q-Lectin			q-35S		
		Ct (Mean ± SD)	DNA/ng/μL (Mean ± SD)		Ct (Mean ± SD)	DNA/ng/μL (Mean ± SD)	
CTAB–NucleoSpin Food Kit method	1	33.89 ± 0.36	0.042	0.023 ± 0.014 ^a^	33.73 ± 0.11	0.053	0.027 ± 0.018 ^a^
	2	35.57 ± 0.08	0.013		35.62 ± 0.19	0.014	
	3	35.39 ± 0.03	0.015		35.47 ± 0.14	0.015	
CTAB-magnetic bead method	1	34.62 ± 0.14	0.025	0.032 ± 0.008 ^a^	34.47 ± 0.07	0.031	0.038 ± 0.012 ^a^
	2	33.83 ± 0.10	0.044		33.66 ± 0.36	0.056	
	3	34.39 ± 0.14	0.029		34.62 ± 0.03	0.028	
CTAB–hexane method	1	/	/	/	/	/	/
	2	/	/		/	/	
	3	/	/		/	/	
CTAB method	1	/	/	/	/	/	/
	2	/	/		/	/	
	3	/	/		/	/	
Resin Based Oil DNA Extraction Kit method	1	/	/	/	/	/	/
	2	/	/		/	/	
	3	/	/		/	/	

Note: Different superscript letters in the same column indicate significant differences among DNA yield (*p* < 0.05); /, no amplification.

**Table 5:** Calibration curves targeting *cp4epsps*.

Target	Standard Curve		Accuracy and Efficiency	
	Slope	Intercept	R^2^	E (%)
q-35S	−3.335	29.365	0.9978	99.46

**Table 6:** Comparison of DNA extraction efficiency based on recoveries.

Methods	Parallel Sample	DNA/ng/μL (Mean ± SD)		DNA Spike-in Quantity/ng	Recovery Rate/% (Mean ± SD)	
CTAB–NucleoSpin Food Kit method	1	6.88	6.96 ± 1.22 ^a^	1120	61.43	62.17 ± 10.91 ^a^
	2	8.50			75.89	
	3	5.51			49.20	
CTAB-magnetic bead method	1	9.31	8.55 ± 0.54 ^a^		83.13	76.37 ± 4.84 ^a^
	2	8.28			73.93	
	3	8.07			72.05	
CTAB–hexane method	1	/	/		/	/
	2	/			/	
	3	/			/	
CTAB method	1	2.21	2.21 ± 1.04 ^b^		19.76	6.69 ± 9 ^b^
	2	/			/	
	3	/			/	
Resin Based Oil DNA Extraction Kit method	1	/	/		/	/
	2	/			/	
	3	/			/	

Note: Different superscript letters in the same column indicate significant differences among DNA yields (*p* < 0.05); /, no amplification.

**Figure A1:**
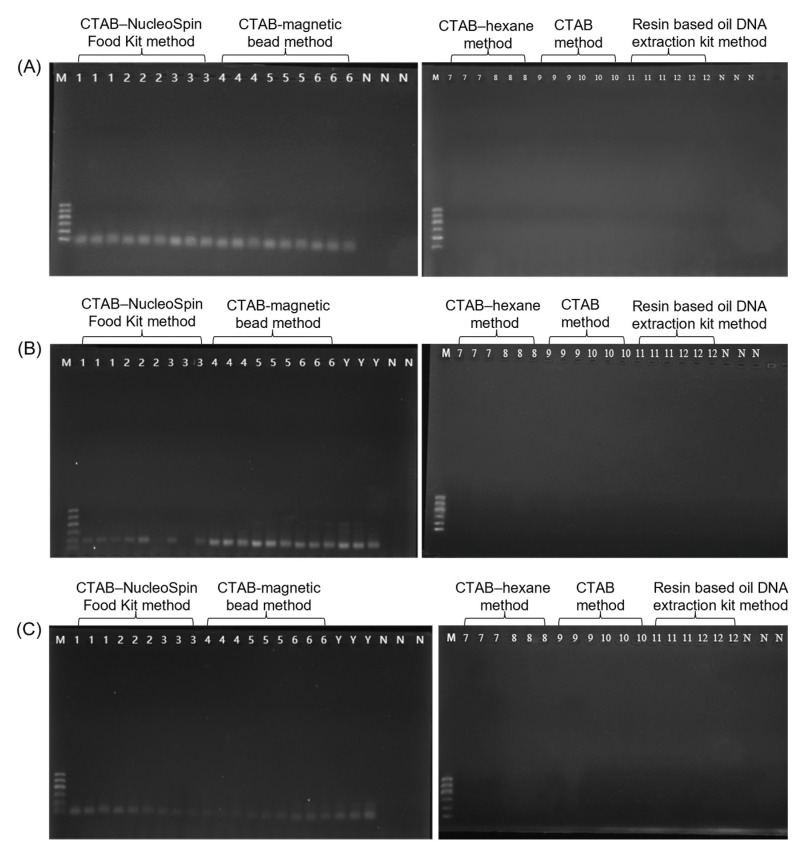
Agarose gel electrophoresis results of different DNA extraction methods based on nested PCR. (**A**) Primer pair-Lectin-F/R1-Lectin-F/R2; (**B**) Primer pair-35S-F/R1- Soybean genome/35S-F/R1; (**C**) Primer pair-35S/CTP-F/R1-35S/CTP-F/R2. Note: M denotes DNA Marker I (TIANGEN, Beijing, China) (100, 200, 300, 400, 500, and 600 bp); Y and N denote positive control and blank, respectively.

## Data Availability

The original contributions presented in the study are included in the article, further inquiries can be directed to the first author.
